# Motivational Determinants of Exercise Behavior in Fitness Centers: Insights from PALMS and Public Health Perspectives

**DOI:** 10.3390/sports14050170

**Published:** 2026-04-23

**Authors:** Bence Cselik, Alexandra Makai, Pongrác Ács, Nikolett Ildikó Tumpek, Gábor László Bátor, Norbert Fóris, Tamás Laczkó, Csilla Filo

**Affiliations:** 1 Physical Activity Research Group, Szentágothai Research Centre, Faculty of Health Sciences, Institute of Physiotherapy and Sport Science, University of Pécs, 7622 Pécs, Hungary; alexandra.makai@etk.pte.hu (A.M.); pongrac.acs@etk.pte.hu (P.Á.); nikolett.tumpek@etk.pte.hu (N.I.T.); csilla.filo@etk.pte.hu (C.F.); 2 Faculty of Health Sciences, Doctoral School of Health Sciences, University of Pécs, 7622 Pécs, Hungary; bator.gabor96@gmail.com (G.L.B.); foris.norbert@gmail.com (N.F.); 3 Faculty of Health Sciences, Institute of Health Insurance, University of Pécs, 7622 Pécs, Hungary; tamas.laczko@etk.pte.hu

**Keywords:** physical activity, exercise behavior, motivation, PALMS, fitness centers, health promotion, public health, BMI, intrinsic motivation, adherence

## Abstract

Regular physical activity is essential for health promotion, yet participation patterns and the motivational determinants of exercise vary widely among recreational exercisers. This study examined exercise behavior and motivational profiles among members of two Hungarian fitness centers using the Physical Activity and Leisure Motivation Scale (PALMS). A cross-sectional survey was completed by 1087 adults, assessing demographics, BMI, exercise frequency, service use, and eight motivational dimensions. Health- and body-related motives were most strongly endorsed, while intrinsic motives (Mastery, Enjoyment) and social or external motives showed greater variability. Exercise frequency was positively associated with Mastery and Competition/Ego motivation, and regression analysis identified Mastery as a significant predictor of attendance, whereas age and female gender were negative predictors. Appearance motivation was positively related to BMI, Affiliation predicted participation in group fitness classes, and Others’ Expectations predicted the use of personal training services. Significant gender- and age-specific motivational differences were also observed. The findings demonstrate that although health and appearance motives dominate, intrinsic and social motives are more strongly linked to sustained engagement. These results highlight the need for motivation-sensitive approaches in fitness programming and public health strategies aimed at promoting long-term exercise adherence and disease prevention.

## 1. Introduction

Regular physical activity plays a central role in maintaining physical and psychological health, contributing to reductions in chronic disease risk, improvements in mental well-being, and enhanced overall quality of life. Nevertheless, global epidemiological data indicate that a substantial proportion of adults fail to meet international physical activity recommendations, highlighting the urgent need to better understand the determinants of sustained exercise participation [1]. Fitness centers have become key environments for structured physical activity, yet long-term adherence often remains low, with many individuals discontinuing regular exercise shortly after initiating a membership [2,3].

A substantial body of evidence indicates that motivational processes fundamentally shape both the initiation and maintenance of exercise behaviors. Self-Determination Theory (SDT) has emerged as one of the most influential frameworks in this domain, emphasizing that the quality of motivation—rather than its sheer quantity—determines whether individuals sustain physical activity over time [4]. SDT posits that autonomous forms of motivation, including intrinsic enjoyment, personal value, and internalized goals, are positively associated with long-term adherence, whereas controlled forms of motivation driven by external pressure or social contingencies are less stable predictors of behavior [5]. Recent theoretical refinements have further underscored that satisfaction of the basic psychological needs for autonomy, competence, and relatedness constitutes a key mechanism by which motivational quality exerts its effects on behavioral persistence [6].

A growing number of empirical studies support the relevance of SDT in explaining why some individuals continue exercising over extended periods while others disengage. For instance, longitudinal research among novice exercisers demonstrates that enjoyment, intrinsic motivation, and competence-related feelings significantly increase the likelihood of maintaining regular attendance at fitness centers [2]. Scoping and systematic reviews further show that SDT-based interventions—particularly those fostering autonomy support, competence reinforcement, and positive interpersonal climates—enhance self-determined motivation and promote sustained engagement in both adolescent and adult populations [7].

Given the theoretical and applied importance of understanding motivational processes, robust measurement tools are essential. The Physical Activity and Leisure Motivation Scale (PALMS) is among the most widely used instruments for capturing multidimensional motives for physical activity. The original validation demonstrated strong internal consistency and an interpretable eight-factor structure comprising mastery, enjoyment, psychological and physical condition, appearance, affiliation, competition/ego, and others’ expectations [8]. Subsequent cross-cultural validations, including the Dutch PALMS [9] and the PALMS-21 among Finnish adolescents [10] confirmed strong psychometric properties, measurement invariance, and predictive validity, reinforcing the scale’s utility in both research and applied fitness contexts. Hungarian validation efforts [11] further highlight the relevance of PALMS for assessing motivational profiles within the local population.

Beyond individual motivational constructs, fitness centers represent complex psychosocial environments in which interpersonal behavior, body-image perceptions, and social influences interact to shape exercise persistence. Evidence suggests that negative body image, perceived judgment, and poorly structured social climates undermine self-determined motivation and may contribute to unhealthy exercise patterns [12,13]. Conversely, autonomy-supportive instructors, positive peer interactions, and high service quality promote psychological need satisfaction and contribute to improved retention [3,14]. Furthermore, structural and logistical factors—such as convenience, institutional support, and environmental barriers—also influence participation patterns, underscoring the need for multidimensional models of exercise adherence [15,16].

Further regional research has also highlighted that substantial differences exist in physical activity patterns across social and demographic groups, which may influence the development of motivational profiles [17]. Objective, accelerometer-based measurements conducted among young adult populations in the Visegrad countries similarly indicate marked heterogeneity in physical activity levels, further reinforcing the need for motivation-sensitive approaches [18]. Studies conducted among university students likewise emphasize the psychosocial determinants of physical activity and their considerable variability [19].

Despite these advances, substantial knowledge gaps remain. Few studies integrate motivational, demographic, psychosocial, and behavioral predictors within large, ecologically valid fitness-center samples. Even fewer examine how multiple motivational dimensions—such as appearance-driven, health-oriented, intrinsic, and social motives—interact to influence exercise frequency, service utilization, and adherence over time. Additionally, limited research has explored how gender-specific socialization and objectification processes may shape motivational trajectories, despite growing evidence that women often begin exercising under external pressures but gradually shift toward more autonomous motives as competence and confidence improve [13].

Further recent research on fitness-center environments also distinguishes between “push” (internal, autonomous) and “pull” (external, environmental, or social) motivational factors, which jointly shape long-term engagement in exercise behavior [20]. This distinction aligns closely with the theoretical framework of Self-Determination Theory and its practical applications, which emphasize that autonomy-supportive environments are most effective for sustaining long-term physical activity [21].

Despite these advances, significant knowledge gaps remain. Few studies have simultaneously examined the multidimensional structure of exercise motivation in fitness-center members using validated frameworks such as the PALMS while integrating demographic and behavioral correlates. Moreover, the translation of specific motivational dimensions into concrete exercise behaviors—such as training frequency or service utilization—has been insufficiently explored. Additionally, the interplay between demographic factors (e.g., age, gender, BMI) and motivational profiles remains underexamined, even though existing evidence suggests that motivational orientations vary substantially across population subgroups.

In light of these gaps, the present study aims to conduct a comprehensive, large-scale investigation of motivational determinants of exercise behavior among fitness-center members. Specifically, the study examines how distinct motivational dimensions relate to exercise frequency, the use of fitness-related services, and demographic characteristics, with the goal of informing motivation-sensitive fitness programming grounded in SDT.

This investigation is guided by the following research questions:

RQ1: How do different motivational dimensions (e.g., intrinsic, appearance-based, social, competence-related motives) relate to exercise frequency among fitness-center members?
RQ2: Which motivational dimensions predict the use of fitness-related services such as group exercise classes or personal training?
RQ3: How are motivational profiles associated with demographic factors including age, gender, and BMI?

Drawing on prior SDT-based research, the study formulated the following hypotheses:

**H1:**  *Higher levels of autonomous motivation (e.g., mastery, enjoyment, physical condition) will be positively associated with greater exercise frequency;* [1,4].

**H2:**  *Socially oriented motivational dimensions (e.g., affiliation, others’ expectations) will predict participation in group-based services and personal training* [3,14].

**H3:**  *Appearance motivation will be positively related to BMI, whereas intrinsic motives will be more prevalent among younger adults* [10,12].

Together, these aims, questions, and hypotheses provide a structured analytic framework that directly addresses the theoretical and empirical gaps identified in the previous literature, positioning the present study to contribute meaningful, evidence-based insights into the motivational determinants of long-term exercise engagement.

## 2. Materials and Methods

### 2.1. Study Design and Setting

This cross-sectional study was conducted in 2025 among members of a private fitness center chain operating in two Hungarian county-level cities. Data collection took place on-site during regular opening hours and was administered using both electronic and paper-based questionnaires to maximize accessibility. Participation was voluntary, anonymous, and did not involve incentives. The study protocol adhered to the principles of the Declaration of Helsinki, and institutional ethical approval was obtained prior to data collection.

### 2.2. Participants

Eligible participants were adults (≥18 years) with an active fitness center membership during the data collection period. Individuals who submitted incomplete questionnaires were excluded from the analysis. Of the 1120 members approached, 1087 provided complete responses and were included in the final sample. The sample consisted of 61.7% women, with a mean age of 29.08 years (SD = 8.4) and a mean BMI of 23.67 kg/m^2^ (SD = 3.9).

### 2.3. Measures

#### 2.3.1. Demographic and Environmental Variables

Participants reported gender, age, height, weight, education level, occupation, and family status. BMI was calculated as weight (kg) divided by height squared (m^2^). Environmental factors included self-reported travel time to the fitness center (<10 min, 10–20 min, 20–30 min, >30 min) and primary point of departure (home, workplace, school).

#### 2.3.2. Exercise Behavior

Exercise frequency was assessed using four categories: daily, 3–4 times per week, 1–2 times per week, and less often. Session duration was categorized as <30 min, 30–60 min, 60–90 min, or >90 min. Additional items assessed preferred gym zones (cardio, strength), use of fitness services (group fitness classes, personal training, sauna, massage), and average weekly training volume. Physical activity was assessed using context-specific questions tailored to fitness center usage patterns (e.g., training frequency, service utilization). While standardized instruments such as IPAQ or the Godin Leisure-Time Exercise Questionnaire provide general estimates of physical activity, they do not capture specific behaviors related to gym-based exercise. Therefore, a targeted measurement approach was considered more appropriate for the aims of this study.

#### 2.3.3. Motivational Assessment (PALMS)

Motivation for exercise was measured using the Physical Activity and Leisure Motivation Scale (PALMS), which consists of 40 items across eight subscales: Mastery, Enjoyment, Psychological Condition, Physical Condition, Appearance, Affiliation, Competition/Ego, and Others’ Expectations. Each item was rated on a 5-point Likert scale (1 = strongly disagree to 5 = strongly agree). Subscale scores were computed as the mean of five items per motivational dimension. The validated Hungarian version of PALMS has demonstrated strong psychometric reliability (Cronbach’s α = 0.85–0.91) [4].

### 2.4. Procedure

Participants were informed about the purpose and voluntary nature of the study. Questionnaires were self-administered and required approximately 10–12 min to complete. Research staff were available to clarify items without providing evaluative feedback. Completed paper-based forms were deposited in sealed collection boxes; online responses were submitted through a secure survey platform. All data were de-identified before analysis and stored in encrypted digital format.

### 2.5. Statistical Analysis

Data analysis was performed using IBM SPSS Statistics (version 29) and Python 3.14 (pandas, scipy, statsmodels). Descriptive statistics were used to summarize demographic characteristics, exercise behavior, and motivational dimensions. Internal consistency of PALMS subscales was assessed using Cronbach’s alpha.

Group differences (gender, age categories, BMI categories, service-use groups) were examined using independent-samples *t*-tests, one-way ANOVA, or chi-square tests, where appropriate. Effect sizes (Cohen’s d, η^2^) were reported to provide information on practical significance.

Associations between motivational dimensions and exercise behavior were evaluated using Pearson or Spearman correlation coefficients, depending on distributional assumptions.

#### 2.5.1. Regression Analyses

Multiple linear regression models were employed to examine predictors of exercise frequency. Logistic regression analyses were used to determine whether motivational dimensions predicted group fitness class participation and personal training use.

Assumptions check: In response to reviewer recommendations, all regression assumptions were formally tested. Residual plots and Shapiro–Wilk tests confirmed the normality of residuals; homoscedasticity was verified using residual-versus-fitted value plots; multicollinearity was assessed using Variance Inflation Factors (VIF < 2.5 for all predictors), indicating no collinearity concerns. Influential outliers were evaluated using Cook’s distance, with no cases exceeding standard thresholds. All models met the required statistical assumptions.

#### 2.5.2. Significance Thresholds

Statistical significance was set at *p* < 0.05 (two-tailed). Following reviewer feedback, non-significant results are no longer described as “trends”; instead, they are explicitly reported as non-significant.

### 2.6. Ethical Considerations

All participants provided informed consent prior to participation. The study adhered to the principles of the Declaration of Helsinki. Data were collected anonymously, handled confidentially, and used solely for research purposes. Institutional ethical approval was obtained from the appropriate review body prior to data collection.

## 3. Results

### 3.1. Sample Characteristics

A total of 1087 participants were included in the final analysis after excluding incomplete responses. Women represented 61.7% of the sample, the mean age was 29.08 years (SD = 8.4), and the mean BMI was 23.67 kg/m^2^ (SD = 3.9). Exercise frequency was generally high: 60.7% reported training 3–4 times per week, followed by 20.1% with 1–2 weekly sessions, 13.0% training daily, and 6.2% exercising less often.

**Figure 1:**
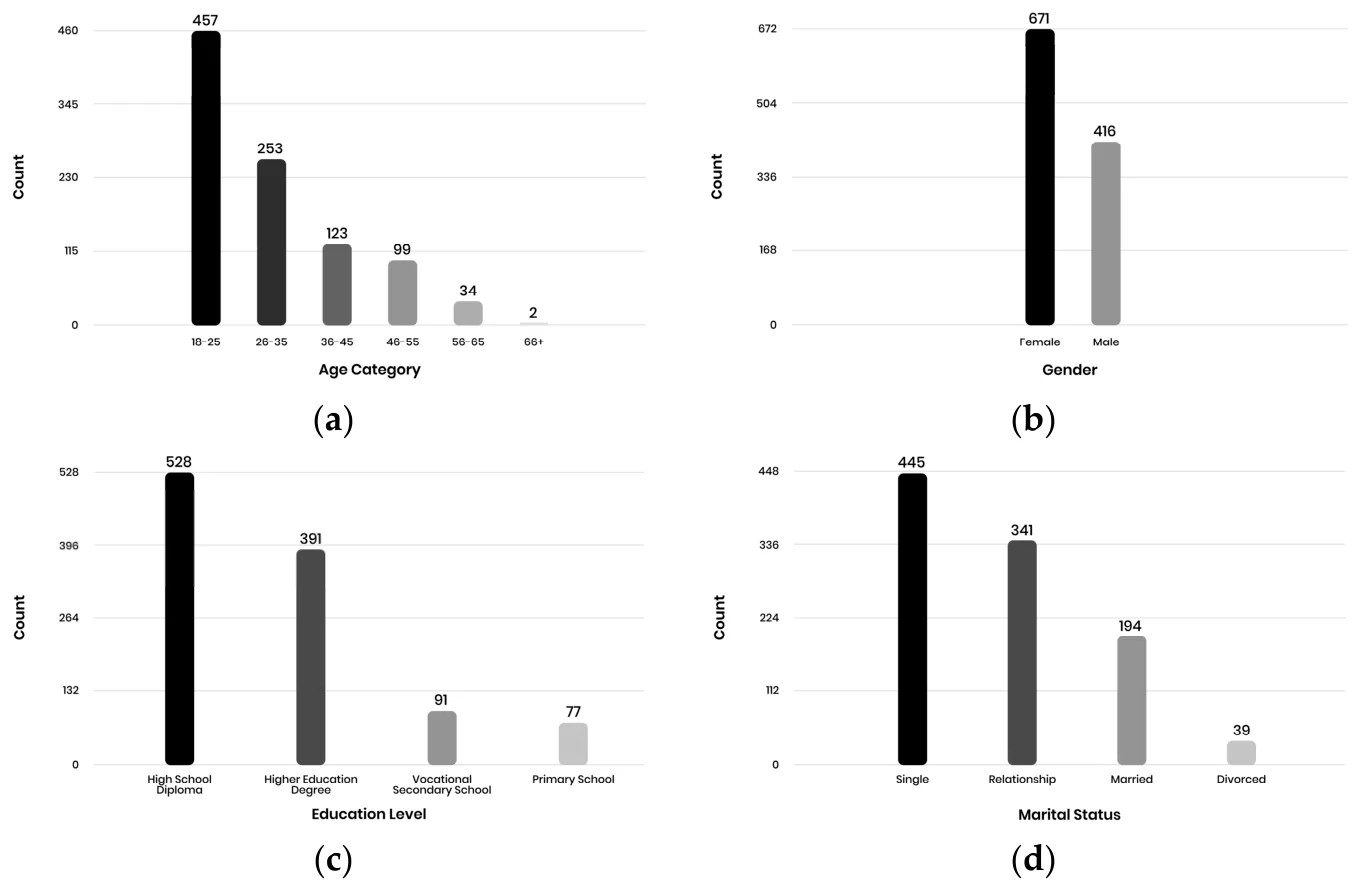
Demographic characteristics of the sample: (**a**) Age distribution, (**b**) Gender distribution, (**c**) Education level, (**d**) Marital status.

The distribution of key demographic variables—including age, gender, BMI categories, education level, and travel time to the fitness center—is visualized in Figure 1, which provides an overview of the composition of the sample and the contextual factors that may shape exercise behavior.

### 3.2. Descriptive Profiles of Motivational Dimensions (PALMS)

Internal consistency of the eight PALMS subscales ranged from acceptable to excellent (Cronbach’s α = 0.63–0.90). Physical Condition (M = 4.51) and Appearance (M = 4.35) emerged as the most highly endorsed motivational dimensions, followed by Psychological Condition (M = 4.20), Enjoyment (M = 4.17), and Mastery (M = 4.10). Lower mean scores were observed for Competition/Ego (M = 2.84), Affiliation (M = 2.80), and Others’ Expectations (M = 1.76).

**Figure 2:**
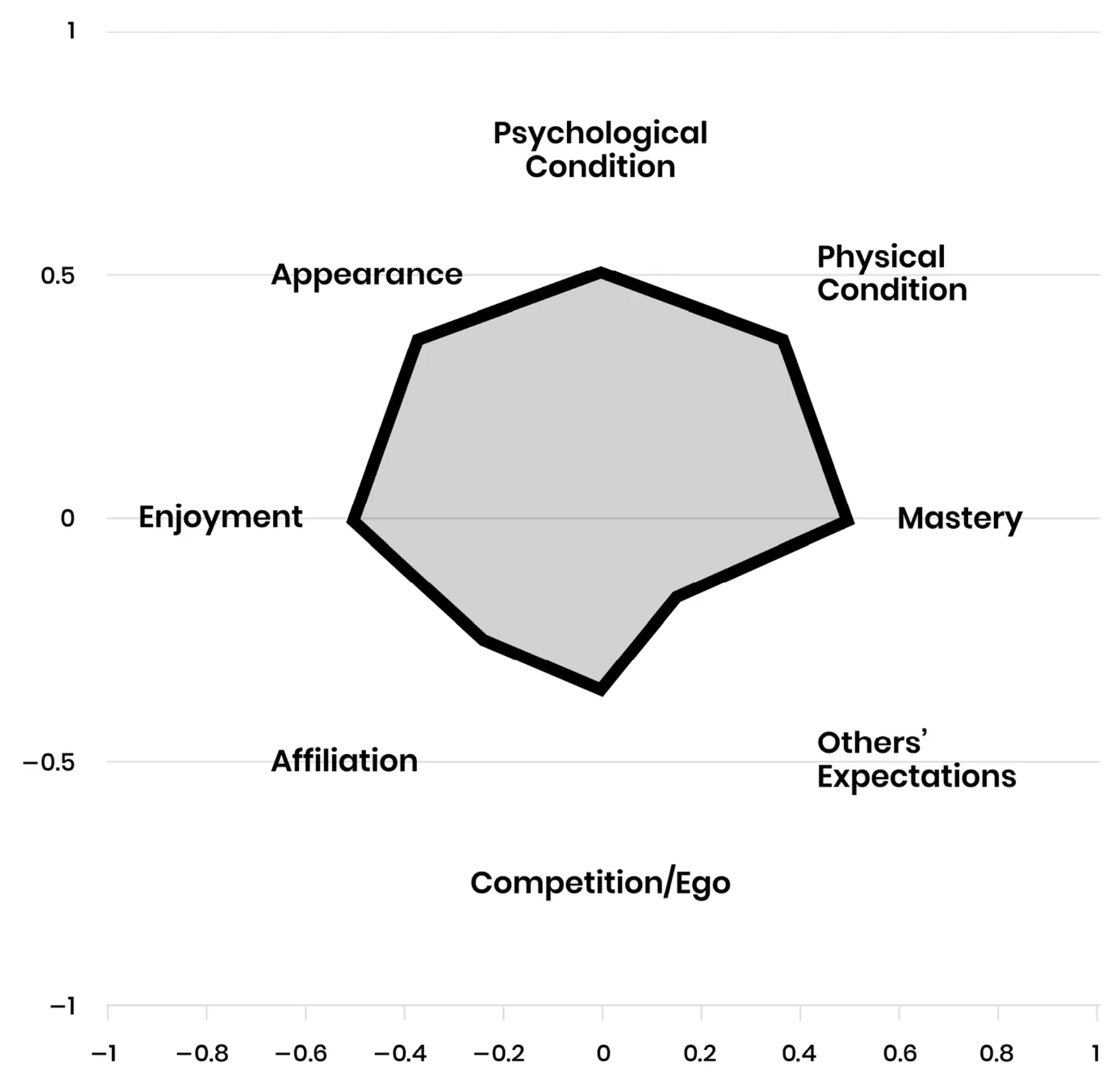
PALMS subscale profile (average scores across eight motivational dimensions).

These overall motivational patterns are visually represented in Figure 2, which illustrates the relative strength of each subscale in the sample.

**Figure 3:**
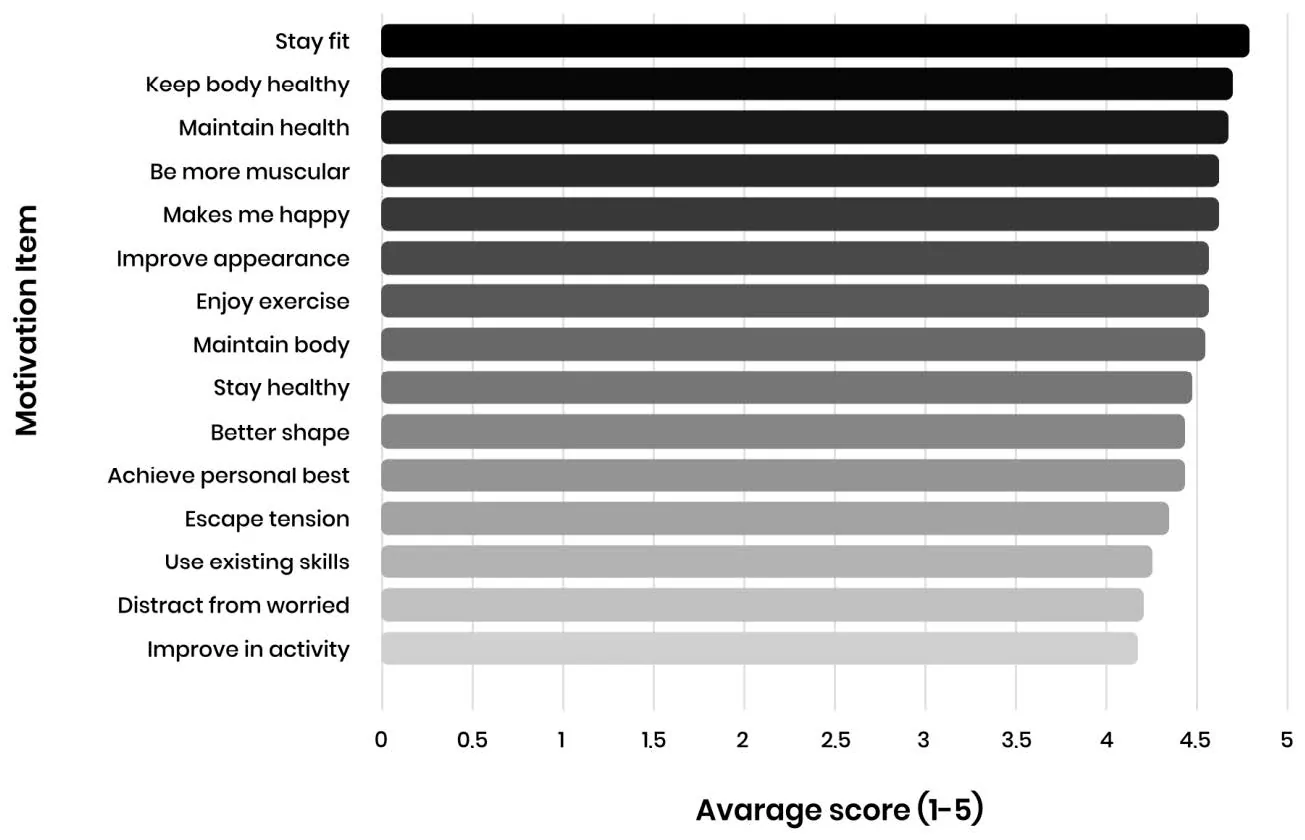
Top 15 motivational items ranked by mean score.

To complement subscale means, the highest-scoring individual motivation items are presented in Figure 3, highlighting that motives related to health, fitness, appearance, and stress reduction dominate participants’ self-reported motivational profiles.

**Figure 4:**
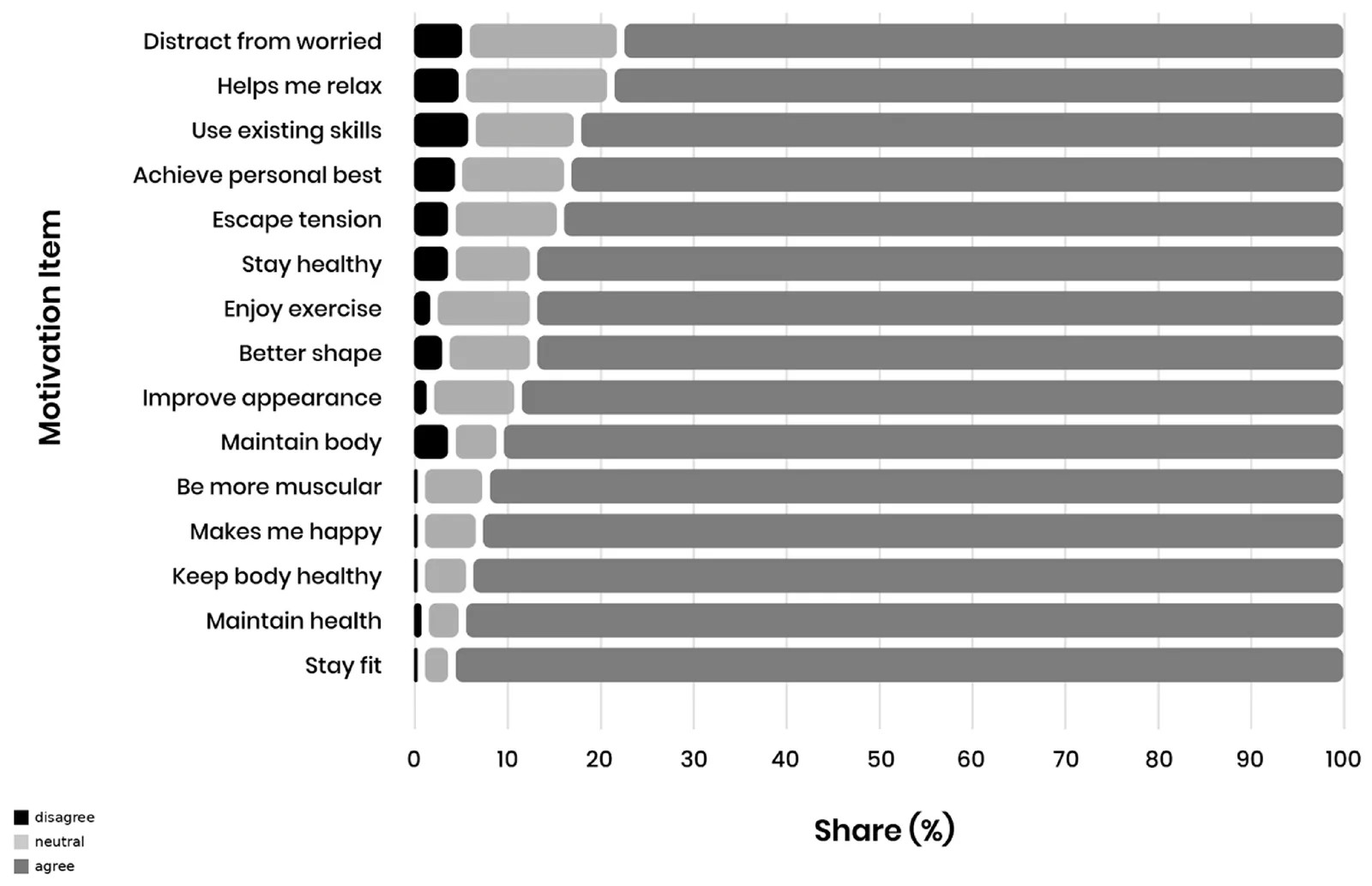
Agreement (Disagree/Neutral/Agree) distribution for the top 15 PALMS motivation items.

Further insight into the attitudinal structure behind these high-scoring items is shown in Figure 4, which provides the share of participants agreeing, responding neutrally, or disagreeing with the most strongly endorsed statements.

### 3.3. Associations Between Motivational Dimensions and Exercise Frequency

Spearman rank correlations indicated positive associations between exercise frequency and Mastery (ρ = 0.110, *p* < 0.001) and Competition/Ego (ρ = 0.191, *p* < 0.001). A weak positive correlation was observed for Enjoyment (ρ = 0.067, *p* = 0.027). Psychological Condition showed a small negative correlation (ρ = −0.075, *p* = 0.014). No significant associations were identified for Physical Condition, Appearance, Affiliation, or Others’ Expectations. Although these correlations were statistically significant, the effect sizes were small, indicating that these motivational dimensions explained only a limited proportion of variance in exercise frequency.

### 3.4. Predictors of Exercise Frequency

Multiple linear regression revealed that Mastery was a significant positive predictor of exercise frequency (β = 0.12, *p* = 0.001). Age (β = −0.006, *p* = 0.002) and female gender (β = −0.227, *p* < 0.001) were significant negative predictors. Travel time was not significantly associated with exercise frequency (*p* = 0.111). (β = −0.0036, *p* = 0.111). Enjoyment and other motives did not significantly contribute to the model.

### 3.5. Relationship Between BMI and Appearance Motivation

Appearance motivation showed a positive correlation with BMI (ρ = 0.193, *p* < 0.001). Regression analysis controlling for age and gender confirmed BMI as a significant predictor (β = 0.022, *p* < 0.001), though the effect size was small. Despite statistical significance, the magnitude of this relationship was small, suggesting limited practical impact.

### 3.6. Social Motivation and Service Utilization

Affiliation motivation significantly differentiated participants who attended group fitness classes from those who did not. Group class participants reported higher Affiliation scores (M = 3.52) compared with nonparticipants (M = 2.65; t = 10.184, *p* < 0.001). Logistic regression demonstrated that each one-point increase in Affiliation nearly doubled the odds of group class participation (β = 0.774, OR ≈ 2.17, *p* < 0.001).

Similarly, Others’ Expectations predicted personal training use (β = 0.368, OR ≈ 1.45, *p* < 0.001). No other motivational dimensions significantly predicted service utilization.

### 3.7. Gender and Age Differences in Motivational Profiles

A multivariate analysis of variance (MANOVA) revealed significant gender and age effects on motivational dimensions (*p* < 0.001). Women scored higher on Appearance, Affiliation, Psychological Condition, and Others’ Expectations, whereas men scored higher on Competition/Ego. Age-related patterns indicated that younger participants (≤24 years) reported higher Mastery, Enjoyment, and Competition/Ego motives, while older adults (≥45 years) scored higher on Physical Condition and Affiliation. Appearance motivation peaked among participants aged 35–44 years.

## 4. Discussion

These findings are consistent with previous work indicating that fitness center members exhibit diverse motivational pathways, and that adherence is strongly shaped by both environmental support and individual motivational quality [2,3]. Research on structured exercise settings further emphasizes that service quality, accessibility, and institutional support are critical determinants of long-term participation, aligning with the present study’s results [15,16].

The present study examined motivational, demographic, and behavioral determinants of exercise engagement among fitness-center members using a multidimensional motivation framework. Overall, the results demonstrate that autonomous motivation—particularly competence-related (Mastery) and intrinsic forms—is central to sustaining regular exercise participation, aligning with Self-Determination Theory (SDT) and prior empirical evidence [1,4]. The findings further support the notion that the quality of motivation exerts greater influence on long-term adherence than the mere presence of motivational drive [5].

Autonomous and competence-related motivation

Mastery emerged as the most robust predictor of exercise frequency, underscoring the importance of competence-supportive motivational structures. This pattern is consistent with SDT’s assertion that need satisfaction drives persistence and well-being [6]. Although Physical Condition and Appearance received the highest mean ratings, their behavioral impact was limited. This dissociation suggests that health- and appearance-oriented motives—often more controlled in nature—may effectively initiate exercise but fail to sustain participation without complementary autonomous motives [1].

The multidimensional pattern observed in this study is also in line with international PALMS validation research, which highlights consistent relationships between intrinsic motives and exercise persistence across cultures [8,9,10]. Hungarian validation studies likewise demonstrate strong psychometric performance and reinforce the scale’s usefulness in distinguishing motivational subprofiles relevant for fitness center participation [11].

Appearance motivation and BMI

The positive association between BMI and Appearance motivation aligns with evidence that individuals with higher BMI often experience heightened body-image pressures, which may trigger controlled motivational orientations [12]. Yet, such motives did not predict exercise frequency, highlighting their instability and limited utility in promoting long-term adherence. Similar patterns have been noted in qualitative work showing that externally driven, appearance-oriented motives are vulnerable to dropout unless they gradually internalize into autonomous forms [13].

Social motives and service utilization

Socially oriented motives—particularly Affiliation—played a crucial role in predicting group fitness class participation. This finding reflects the importance of relatedness as a core psychological need and is consistent with literature showing that social environments can strengthen motivation and adherence [14]. The association between Others’ Expectations and personal-training use indicates that external regulatory processes may drive individuals toward more structured, supervised forms of exercise. Taken together, these results suggest that fitness-center services serve distinct motivational functions: group exercise satisfies social-connection needs, whereas personal training provides accountability and structure for individuals with more externally anchored motives.

Recent work in fitness settings also distinguishes between “push” (internal, autonomous) and “pull” (external or environmental) motivational factors, offering a nuanced framework for interpreting why some participants rely more heavily on social or externally regulated motives [20]. This framework aligns directly with SDT-based applications showing that autonomy-supportive environments enhance the internalization of motives and encourage long-term adherence [21].

Demographic variation in motivational profiles

Gender and age differences observed in this study parallel known motivational patterns. Women demonstrated higher Appearance, Affiliation, and emotion-related motives, a pattern consistent with findings linking female exercisers’ motivation to sociocultural pressures and relational factors [13]. Younger exercisers reported stronger intrinsic and performance-oriented motives, whereas older participants prioritized health- and connection-oriented motives, consistent with age-related motivational shifts documented in population-level studies [7]. These variations underline the importance of tailoring intervention strategies to demographic subgroups.

Regional studies further suggest that demographic and social-group differences strongly influence physical activity patterns, which may help explain the motivational differences observed in the present sample [17]. Objective accelerometer-based research from Visegrad countries likewise shows substantial variability in activity levels among young adults, underscoring the need to consider broader behavioral and environmental contexts [18]. Similar variability is noted in lifestyle studies conducted among university students, emphasizing the role of psychosocial determinants in shaping exercise engagement [19].

Implications for fitness-center programming

The results emphasize that competence-supportive environments—featuring structured skill development, feedback, and progression opportunities—may be particularly effective in fostering persistent engagement. Socially oriented exercisers may benefit from group-based formats that strengthen relational ties, while individuals driven by external expectations may require guided support to facilitate the internalization of motives. Given the limited behavioral influence of appearance-based motives, fitness-center messaging should shift away from appearance-focused communication toward competence, well-being, and intrinsic enjoyment.

Public-health and industry implications

The findings have broader implications for health promotion. Interventions that cultivate autonomous forms of exercise motivation and satisfy basic psychological needs may generate more durable behavioral change than those emphasizing risk reduction or appearance modification. As fitness centers increasingly serve as primary sites for recreational exercise, understanding the motivational determinants of engagement becomes vital for designing environments capable of promoting sustained public-health benefits.

Service Utilization Patterns in Fitness Center Settings

The association between the use of personal training services and the “Others’ Expectations” motivational dimension can be understood through the role of external regulation and social pressures. Empirical evidence shows that external, introjected, or expectation-driven forms of motivation are closely linked to higher levels of social physique anxiety, which in turn increase individuals’ preference for structured, supervised forms of exercise [22]. Recent findings indicate that external and introjected regulation are positively associated with social physique anxiety, suggesting that individuals with stronger externally regulated motives may be more inclined to seek accountability-oriented services such as personal training.

## 5. Conclusions

This study demonstrates that although health- and appearance-related motives are the most strongly endorsed among fitness center members, sustained exercise behavior is more closely linked to intrinsic and social motivational dimensions. Mastery emerged as the strongest positive predictor of exercise frequency, whereas age and female gender were negative predictors. Socially oriented motives showed distinct behavioral effects: Affiliation substantially increased the likelihood of participating in group fitness classes, while Others’ Expectations predicted personal training use. Appearance motivation displayed a small but significant positive association with BMI, highlighting its relevance particularly for individuals with higher body weight.

Overall, the findings underscore that exercise motivation is multidimensional, and that motives differ not only in their content but also in their behavioral impact. Strategies that strengthen competence, support interpersonal connectedness, and provide structured accountability are likely to be more effective for promoting long-term adherence than approaches relying solely on health- or appearance-based messaging. Understanding motivational profiles can therefore play an essential role in designing exercise programs and public health interventions that foster sustainable engagement in physical activity.

### 5.1. Strengths and Limitations

A major strength of this study is its large sample size (*N* = 1087), which enhances statistical power and allows for robust subgroup analyses across gender, age, BMI categories, and motivational profiles. The use of the validated Hungarian version of the Physical Activity and Leisure Motivation Scale (PALMS) provides a comprehensive and psychometrically sound assessment of eight motivational dimensions, enabling a multidimensional examination of exercise behavior. The study also benefits from its focus on a real-world fitness center environment, offering ecologically valid insights into the motivational characteristics of recreational exercisers. Additionally, the integration of both behavioral (exercise frequency, service use) and psychological (motivation) variables provides a comprehensive understanding of the determinants of fitness center engagement.

However, several limitations should be acknowledged. First, the cross-sectional design prevents causal inferences about the directionality of relationships between motivational factors and exercise behavior. Second, all measures—including exercise frequency, BMI, and motivational constructs—were self-reported, which may introduce recall bias and social desirability bias. Third, the sample was drawn from two urban fitness centers, which may limit generalizability to rural populations, different socioeconomic groups, or individuals who exercise outside gym-based environments. The motivational patterns identified here may not fully represent inactive or beginner populations. Finally, although PALMS captures key motivational dimensions, it does not account for contextual factors such as facility culture, instructor behavior, or digital engagement, which could further influence adherence. The findings of this study should be interpreted with caution. While motivational factors appear to be associated with exercise behavior, the cross-sectional nature of the study does not allow for causal conclusions.

Future research should consider longitudinal designs, the incorporation of objective physical activity measures (e.g., accelerometry), and a broader range of fitness environments to enhance generalizability and deepen understanding of motivational dynamics over time.

### 5.2. Practical Implications

The findings of this study offer several practical directions for fitness center programming, coaching practices, and public health interventions:

### 5.3. Program Design and Fitness Center Operations

Strengthen competence-oriented pathways: Providing progressive training plans, technique instruction, and performance feedback can support mastery-oriented individuals and enhance long-term engagement.

Support social connectedness: Expanding group fitness options, creating community events, and implementing buddy or small-group systems may increase adherence, particularly among individuals with high Affiliation motivation.

Provide structured accountability for externally motivated individuals: Regular trainer check-ins, app-based reminders, and personalized follow-ups may be effective for those who score high on Others’ Expectations.

### 5.4. Coaching and Instruction

Use mastery-centered feedback: Emphasize measurable progress, skill development, and personal improvement over appearance-related messaging.

Foster positive group dynamics: Encourage cooperative goals, social interaction, and supportive atmospheres within group classes to reinforce belonging.

### 5.5. Tailoring to Demographic Differences

Younger adults: Offer performance-oriented challenges and skill-based progression.

Older adults: Prioritize functional health, stress reduction, and supportive social environments.

Women: Highlight psychological well-being, stress management, and social connection.

Men: Integrate competitive or performance-driven elements.

### 5.6. Public Health and Institutional Applications

Motivation-sensitive health promotion: Messaging that builds competence, enjoyment, and social engagement may support adherence more effectively than appearance- or risk-based approaches.

Enhancing accessibility: Flexible membership options, short-format sessions, and well-timed classes can reduce logistical barriers and encourage participation among less active individuals.

Monitoring motivational changes: Brief motivational assessments (e.g., PALMS-based screens) at onboarding and periodic follow-ups may help tailor interventions over time.

Together, these implications highlight the value of aligning exercise programming with the specific motivational drivers of different population groups to improve adherence and support long-term health outcomes.

## Data Availability

The original contributions presented in this study are included in the article. Further inquiries can be directed to the corresponding author.

## Abbreviations

The following abbreviations are used in this manuscript:

PALMS	Physical Activity and Leisure Motivation Scale
SD	Spread
BMI	Body Mass Index
KG	Kilogram
MANOVA	Multivariate analysis of variance
MIN	Minute
ANOVA	Analysis of variance

## References

[1] Teixeira P.J., Carraça E.V., Markland D., Silva M.N., Ryan R.M. (2012). Exercise, physical activity, and self-determination theory: A systematic review. Int. J. Behav. Nutr. Phys. Act..

[2] Gjestvang C., Abrahamsen F.E., Stensrud T., Haakstad L.A.H. (2020). Motives and barriers to initiation and sustained exercise adherence in a fitness club setting: A one-year follow-up study. Scand. J. Med. Sci. Sports.

[3] Yeomans C., Karg A., Nguyen J., McDonald H. (2023). Attitudinal and behavioural determinants of fitness centre member retention. Manag. Sport Leis..

[4] Wilson P.M., Mack D.E., Grattan K.P. (2008). Understanding motivation for exercise: A self-determination theory perspective. Can. Psychol..

[5] Ntoumanis N., Moller A.C. (2025). Self-determination theory informed research for promoting physical activity: Contributions, debates, and future directions. Psychol. Sport Exerc..

[6] Wang J.C.K., Hagger M.S. (2023). Self-Determination Theory in Physical Activity Contexts. The Oxford Handbook of Self-Determination Theory.

[7] Barbosa Cano D., Gomez-Baya D. (2025). SDT-based interventions to promote physical activity in adolescents: A scoping review. Youth.

[8] Molanorouzi K., Khoo S., Morris T. (2014). Validating the physical activity and leisure motivation scale (PALMS). BMC Public Health.

[9] van Lankveld W., Linskens F., Stolwijk N. (2021). Motivation for Physical Activity: Validation of the Dutch Version of PALMS. Int. J. Environ. Res. Public Health.

[10] Ruiz M.C., Hassan M., Aypar E., Knittle K., Tolvanen A., Robazza C. (2025). Validation of the 21-item PALMS among Finnish late adolescents. Psychol. Health.

[11] Kovács K., Boda-Ujlaky J.E., Török L., Dömsödi R., Gyömbér N. (2024). A Szabadidős Testedzés Motivációja Skála (PALMS–H) pszichometriai vizsgálata. Mentálhigiéné Pszichoszomatika.

[12] Salvador R., Monteiro D., Rebelo-Gonçalves R., Jiménez-Castuera R. (2023). Interpersonal behavior, basic psychological needs, motivation, eating behavior, and body image in gym exercisers. Sustainability.

[13] Deininger C., Teriba A.A., Foley-Nicpon M. (2025). Objectification and self-determination in fitness: A qualitative investigation of women’s motivations for physical exercise. Soc. Sci..

[14] Marques P., Machado S., Teixeira D. (2025). Psychological determinants of exercise practice in health clubs. Cuad. Psicol. Deporte.

[15] Schroeder E.C., Gregory J., Welk G.J., Franke W.D., Lee D. (2017). The association of health club membership with physical activity and cardiovascular health indicators. PLoS ONE.

[16] Huang W., Lai D., Zeng M., Chen B., Ye S., Li F., Huang C. (2025). Association of leisure-time aerobic physical activity time with apparent treatment-resistant hypertension: A study based on NHANES database. Biol. Sport.

[17] Puszczałowska-Lizis E., Lizis S., Głód P., Kitschke E. (2025). Health-promoting behaviors of women and their opinions on the quality of fitness services, depending on experienced musculoskeletal injuries. Health Probl. Civiliz..

[18] Stelmach M.J., Baj-Korpak J., Weiner M., Niźnikowska E.A., Ács P., Salonna F., Buková A., Hajduchová H., Šedová L. (2026). Accelerometer-derived physical activity and health correlates among students in the Visegrad Group countries. Health Probl. Civiliz..

[19] Junger J., Hrobacz A., Melichar R., Salonna F. (2025). Physical activity and sedentary behavior in the lifestyle of university students. Health Probl. Civiliz..

[20] Doğan M., Sevilmiş A., Nalbant U. (2025). The Impact of Push and Pull Motivational Factors on Fitness Members’ Satisfaction and Behavioral Intentions. Research Square.

[21] Quested E., Kritz M., Hancox J.E., Ntoumanis N., Thøgersen-Ntoumani C., Zenko Z., Jones L. (2021). Promoting self-determined motivation for physical activity: From theory to intervention work. Essentials of Exercise and Sport Psychology.

[22] Macila E., Dogan E., Sancar N. (2024). Investigating Correlation between Exercise Participation Motivation and Social Physique Anxiety and Their Differences across the Exercise Stages of Change. Sports.

